# Assessment of Immunological Biomarkers in the First Year after Heart Transplantation

**DOI:** 10.1155/2015/678061

**Published:** 2015-09-30

**Authors:** Maja-Theresa Dieterlen, Katja John, Hartmuth B. Bittner, Meinhard Mende, Attila Tarnok, Friedrich W. Mohr, Markus J. Barten

**Affiliations:** ^1^Clinic for Cardiac Surgery, Heart Center Leipzig, University Hospital Leipzig, 04289 Leipzig, Germany; ^2^Division of Thoracic Transplantation, Florida Hospital Orlando, Orlando, FL 32803, USA; ^3^Clinical Trial Centre Leipzig, University of Leipzig, 04103 Leipzig, Germany; ^4^Department of Pediatric Cardiology, Heart Center Leipzig, University Hospital Leipzig, 04289 Leipzig, Germany; ^5^Department of Cardiovascular Surgery, University Heart Center Hamburg, 20246 Hamburg, Germany

## Abstract

*Background*. Pharmacodynamic biomarkers that detect changes of immunological functions have been recognized as a helpful tool to increase the efficacy of immunosuppressive drug therapies. However, physiological changes of immunological biomarkers following transplantation are not investigated. Therefore, we assessed frequently used immunological biomarkers of the circulating blood in the first year following heart transplantation (HTx). *Methods*. Activation markers CD25 and CD95, intracellular cytokines IL-2 and IFN*γ*, chemokines IP10 and MIG, and subsets of dendritic cells as well as antibodies against human leukocyte antigens (HLA) and major histocompatibility complex class I-related chain A (MICA) antigens were analyzed at different time points using flow cytometry and Luminex xMAP technology. *Results*. Expression of IL-2, IFN*γ*, and plasmacytoid dendritic cells (pDCs) significantly increased (*p* < 0.01) during the first year. Anti-HLA antibodies decreased continuously, while anti-MICA antibodies showed minor increase within the first year. An association between percentage of pDCs and anti-MICA antibody positivity was proven. pDCs, IFN*γ*-producing T cells, and IP10 concentration were associated in a stronger way with age and gender of HTx recipients than with antibodies against HLA or MICA. *Conclusions*. We conclude that certain immunological biomarkers of the circulating blood change during the first year after HTx. These changes should be considered for interpretation of biomarkers after transplantation.

## 1. Introduction

Predictive biomarkers play a major role in identifying transplant rejection following solid organ transplantation. Especially after heart transplantation (HTx), the first year is critical for transplant survival and long-term outcome. The post-HTx follow-up care includes the pharmacokinetic control of immunosuppressive drug blood levels as well as histopathological analyses of endomyocardial biopsies (EMBs) in periodical intervals. However, this strategy does not avoid over- or underimmunosuppression which on their part often leads to severe infection or allograft rejection, respectively.

Different patient-specific responses of the immune system seem to be the reason for the variable efficacy of immunosuppressive drug therapies. The reason for the variable efficacy of immunosuppressive drug therapies is due to the different, patient-specific response of the immune system. For an enhanced estimation of the effectiveness of immunosuppressive drug therapies, pharmacodynamic monitoring becomes more and more important. The clinical research in the last decade focused on the identification of biomarkers which are detectable in a noninvasive or minimally invasive way, for example, in biological fluids like blood, serum, or urine. Accordingly, a number of candidate immunological biomarkers have been investigated to find a predictive marker for an enhanced pharmacodynamic and transplant rejection monitoring, respectively. For example, the acquisition of the activation status of T cells by measuring surface molecules such as CD25 or CD95 is one of the major starting points of immune monitoring [1]. Analysis of intracellular cytokines of T cells like interleukin-2 (IL-2) and interferon-*γ* (IFN*γ*) or downstream chemokines of IFN*γ*-signalling like CXC chemokine ligand 9 (CXCL9; MIG) and CXC chemokine ligand 10 (CXCL10; IP10) indicated that they have been identified as potential candidate biomarkers which are able to correlate with graft outcome [2, 3]. As regulators of immune reactivity, plasmacytoid and myeloid dendritic cells (pDCs and mDCs) predict outcomes after different organ transplantation due to their ability to detect acute cellular rejection (ACR), long-term graft loss, and infectious complications and to reflect the degree of immunosuppression [4].

Humoral processes in terms of antibody-mediated rejection (AMR) could be involved in the development of hyperacute, acute, and chronic allograft dysfunction. Specifically, antibodies (Abs) against human leukocyte antigens (HLA) class I and class II or against major histocompatibility complex class I-related chain A (MICA) have been associated with reduced allograft survival and rejection following HTx [5–7].

As mentioned above, it has to be considered that immune system pathways and interplays as well as immunomodulatory molecules can vary strongly in the first year following transplantation. Thus, we hypothesize that immunological markers could vary in the first year after transplantation. To investigate the physiological changes of immunological biomarkers following transplantation, the present study characterized frequently used immunological biomarkers of the circulating blood after HTx.

## 2. Materials and Methods

### 2.1. Sample Collection

Heparinized whole blood samples and sera from 46 consecutively heart transplanted recipients were collected within the first year after transplantation at different time points: (1) within the first month, (2) in the second to third month, (3) in the fifth to sixth month, (4) in the eighth to ninth month, and (5) in the eleventh to twelfth month. Whole blood was used for flow cytometric analysis. Sera were aliquoted and stored at −80°C until multiplex screenings. The study was approved by the Ethic Committee of the Medical Faculty of the Leipzig University (number 123-11-18042011, date of approval 18 April 2011). The subjects gave written informed consent.

### 2.2. Histopathological Analysis of EMBs

Normal biopsy schedule in the first year after HTx was monthly for the first six months and every three months in the second half year. The right internal jugular veins or the right femoral veins were used for approach. After local anesthesia with 2% lidocaine the vein puncture was performed through insertion of a 7-French sheath in Seldinger technique. Under X-ray control, special biopsy forceps were used to harvest five fragments of myocardial tissue from the apical segment of the right side of the interventricular septum. After removal of biopsy forceps, area of puncture was compressed for 5 min. Harvested myocardial tissue pieces were stored in 4% formaldehyde-PBS until paraffin embedding and histological section cutting. Histopathological evaluation of EMBs was performed by staining with haematoxylin-eosin and grading according to International Society for Heart and Lung Transplantation (ISHLT) nomenclature adopted in 1990 [8].

### 2.3. Flow Cytometry

Flow cytometric data were acquired and analyzed using a* BD LSR II Flow Cytometer* (BD, Heidelberg, Germany) and* BD FACSDiva version 6.1.3* software. All Abs were obtained from BD. Isotype controls and fluorescence-minus-one (FMO) controls were used to identify gating boundaries and autofluorescence staining and acquisition of CD25, CD95, and dendritic cell (DC) subsets was performed as described previously [9, 10]. In brief, the simultaneous detection of the T cell activation markers CD25 and CD95 was performed by stimulating heparinized whole blood samples with 4.5 *μ*g/mL phytohemagglutinin-L (*PHA*-L) for 48 h at 37°C, 5% CO_2_, and 95% relative humidity. Cells were trypsinized with trypsin EDTA (1) 0.05%/0.02% in DPBS (PAA Laboratories GmbH, Pasching, Austria) for a maximum of 10 min at 37°C followed by washing with phosphate-buffered saline (PBS) (Biochrom GmbH, Berlin, Germany) and centrifugation at 300 ×g for 5 min. Cells were resuspended in 2 mL PBS. 200 *μ*L of this cell suspension was stained for 30 min using mouse anti-human CD25-PE, mouse anti-human CD95-FITC, and mouse anti-human CD3-PECy5. Then, cells were washed, lysed with BD lysis buffer (BD), fixed with 500 *μ*L 1% formaldehyde-PBS, and stored at 4°C until analysis. 10,000 CD3-positive events were analysed.

Dendritic cell subsets were stained by using 300 *μ*L heparinized whole blood and an antibody cocktail containing mouse anti-human lineage cocktail-1-FITC, mouse anti-human CD123-PECy5, mouse anti-human CD11c, and mouse anti-human HLA-DR-PE. The cell-antibody mixture was incubated for 20 min at room temperature in dark. Cells were then mixed and treated with 2 mL BD lysis buffer for 10 min at room temperature. Following lysis, cells were washed, fixed with 1% formaldehyde-PBS, and stored at 4°C until analysis. 500,000 events were analyzed per sample.

For the detection of the intracellular cytokines heparinized whole blood was stimulated for 30 min at 37°C using 25 ng phorbol-myristate-acetate and 15 ng ionomycin (both from Sigma-Aldrich Chemie GmbH, Taufkirchen, Germany) per mL blood. 10 *μ*g of Brefeldin A (Sigma) was added for 5 hrs at 37°C to induce the accumulation of the intracellular cytokines. Subsequently, 50 *μ*L of stimulated whole blood was stained with 5 *μ*L of mouse anti-human CD3-PECy5. IntraPrep Permeabilization Reagent (Beckman Coulter, Krefeld, Germany) was used to permeabilize blood cells. Then, mouse anti-human IFN*γ*-FITC and mouse anti-human IL2-APC antibodies were used to stain intracellular cytokines. 10,000 CD3-positive events were analyzed.

### 2.4. Multiplex Analysis

Multiplex analysis was performed on* Luminex 100 IS System* (Luminex, Austin, TX, USA) and with* Luminex 100 IS 2.3* and* HLA Fusion 2.0* software. Chemokine levels of MIG (CXCL9) and IP10 (CXCL10) were quantified in serum samples using* Human Singleplex Bead Kits* (Life Technologies GmbH, Darmstadt, Germany) according to manufacturer's instructions. Sera were screened for Abs against HLA class I, HLA class II, and MICA with LABScreen mixed class I, class II, and MICA (BMT GmbH, Meerbusch-Osterath, Germany) according to manufacturers' instructions. Acquisition and analysis were performed with Luminex 200 and* HLA Fusion Software*. Cutoff value for positive results was set to 3.0.

### 2.5. Statistical Analysis

Patient cohort was characterized by mean (±standard deviation) for continuous variables and by number (percent) for categorical variables. Firstly, courses analysis of the biomarkers over five time points was performed. By reason of a number of missing values the time points were reduced to three (1st quarter, 2nd quarter, and 3rd/4th quarter corresponding to 2nd half year) and every value was assigned to the nearest. Secondly, missing values were imputed by multiple imputations by chained equations [11] for construction of a combined set. On the basis of this complete set the time courses were analyzed by means of ANOVA models with repeated measurements including adjustment of *F*-statistics and correction of degrees of freedom. If the test of inner-subject effect “time” was significant, pairwise contrasts were tested including Bonferroni-Holm adjustment [12].

GEE (general estimating equations) were used for analysis of biomarkers with different Ab status in a multivariate model with gender and age as covariates. Tests were performed two-sided at a 5% significance level. All analyses were done by* Intel SPSS Statistics version 20* (© IBM Corp. 1989, 2011).

## 3. Results

### 3.1. Patients

Demographic data and data on transplantation history are shown in Table 1. Forty-six HTx recipients were included in this study with a mean age at HTx of 50.6 ± 12.5 yrs. Thirteen (28%) recipients were female. Initial immunosuppressive triple therapy consisted of tacrolimus, mycophenolate mofetil, and steroids. Two HTx recipients (4.3%) died within the first year after transplantation. Five recipients were bridged to transplantation with assist devices, whereas the mean assist device support was 21 months, and assist device support duration ranged from 11 to 35 months. One patient included in this study was retransplanted, whereas the lifespan of the first organ was 16.8 years. Retransplantation was necessary because of a severe 3-vessel cardiac allograft vasculopathy. Nine HTx recipients (19.6%) developed mild cellular rejections (grade IB) within the first year that were detected by histological analysis of EMB. Six of these nine recipients (13.0%) developed a rejection in the first three months of the first year. To avoid a biased comparison of immunological markers in patients with and without cellular rejections, only patients with rejection in the first three months and patients without cellular rejection were analysed (Table 1).

### 3.2. Biomarker Stability during the First Year after HTx

No significant changes were observed for the markers CD25, CD95, IP10, MIG, and mDCs during the first year after HTx. However, significant changes (*p* < 0.01 for all three markers) of IL-2- and IFN*γ*-positive T cells as well as of the percentage of pDCs could be detected in the first year. All three markers showed significant increases during the observation period (Table 2).

**Figure 1:**
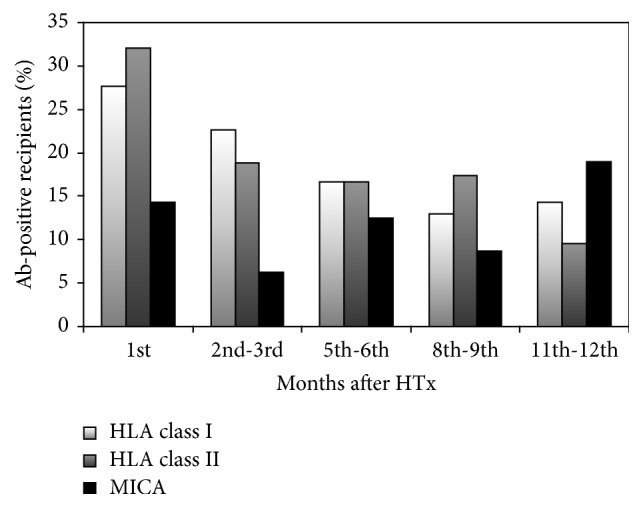
Percentage of recipients with positive Ab status for HLA class I-, HLA class II-, and MICA-Abs during the first year after HTx. Ab, antibody; HLA, human leukocyte antigen; HTx, heart transplantation; MICA, major histocompatibility complex class I-related chain A.

The percentage of recipients with positive Ab status for anti-HLA class I- and class II-Abs decreased continuously during the first year from 27.6% anti-HLA class I-Ab-positive and 32.1% anti-HLA class II-Ab-positive recipients in the first month after HTx to 14.3% anti-HLA class I-Ab-positive and 9.5% anti-HLA class II-Ab-positive recipients at the end of the first year after HTx (Figure 1). The percentage of anti-MICA-Abs alternated during the observation period. Comparison of the percentage of recipients with positive anti-MICA-Ab status from 1st month to 12th month after HTx showed a minor increase from 14.3% in the 1st month to 19.0% in the 12th month (Figure 1).

### 3.3. HLA and MICA Sensitization Analysis

Complex statistical analysis using GEE showed a relationship between MICA positivity and percentage of pDCs (*p* < 0.01). In the first six months after transplantation pDCs were lower in MICA-positive recipients (1st quarter: 16.3%  ± 10.6%; 2nd quarter: 24.1%  ± 11.0%) compared to MICA-negative recipients (1st quarter: 41.6%  ± 9.3%; 2nd quarter: 34.0%  ± 9.0%). Further analysis revealed that pDCs, IFN*γ*-producing T cells, and IP10 sera concentration were associated in a stronger way with age and gender of HTx recipients than with Abs against HLA class I, HLA class II, or MICA.

### 3.4. Comparison of Immunological Biomarkers in Recipients with and without Mild Cellular Rejections

**Figure 2:**
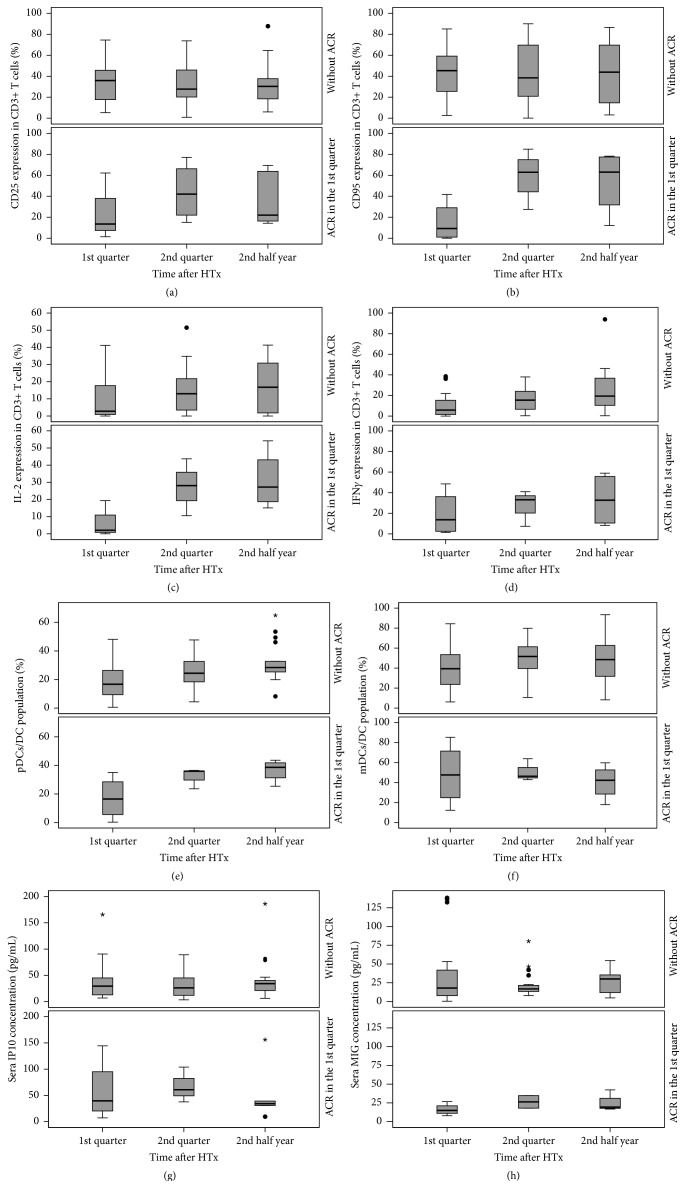
Chronological sequence of cellular parameters and cytokines within the first year after HTx in recipients with and without acute cellular rejections. The activation markers CD25 (a) and CD95 (b), the intracellular cytokines interleukin-2 (c) and interferon-*γ* (d), and percentages of plasmacytoid dendritic cells (e) and myeloid dendritic cells (f) on whole DC population as well as sera concentrations of peripheral chemokines IP10 (g) and MIG (h) were shown. ACR, acute cellular rejection; DC, dendritic cell; IP10, CXC chemokine ligand 10; IFN*γ*, interferon-*γ*; IL-2, interleukin-2; mDCs, myeloid dendritic cells; MIG, CXC chemokine ligand 9; pDCs, plasmacytoid dendritic cells.

Statistical analysis of biomarker expression in recipients with mild cellular rejection in the first three months (*n* = 6) and in recipients without rejection in the first year (*n* = 37) revealed that the discrimination between biomarker profiles of recipients with and without ACR is impeded by the high range of each single immunological marker (Figure 2). Significant differences between recipients with and without ACR could be observed for CD95-positive T cells in the first quarter (*p* = 0.033) and for IP10 sera concentration in the second quarter of the first year (*p* = 0.017) (Table 3).

## 4. Discussion

In our study, analysis of biomarker validity showed that percentage of IL-2- and IFN*γ*-producing T cells as well as the percentage of pDCs changed during the first year after HTx while the other tested biomarkers did not change significantly. These data implicate that the time point of blood withdrawal may play an important role in data interpretation. The stability of the used biomarkers was not investigated in previous studies. In general, we recommend the investigation of biomarker profile after transplantation to prove robustness and reliability of these biomarkers. Heidt et al. [13] advised investigating differentiation capacity between immune activation toward the allograft and infectious agents for any biomarker to be clinically applicable for predicting rejection.

In the early posttransplantation period, AMR may cause serious complications or allograft failure. EMB histology is not predictive for AMR, but it is assumed that anti-HLA- and anti-MICA-Abs are associated with high risk for AMR [7, 14]. Our data showed that Abs against HLA class I and class II decreased continuously after HTx. This effect may be caused by the immunosuppressive regime that affects B cells, thereby influencing Ab production of these cells. In contrast, the percentage of recipients with anti-MICA-Abs slightly increased during the first year after HTx. Exclusively, ongoing monitoring will show if* de novo* production of anti-HLA- and anti-MICA-Abs leads to complications after HTx.

One limitation of our study was the low number of recipients with ACR in the first three months and the absence of recipients with clinically manifested ACR confirmed by EMB with histological grading of grade 2A or higher. Statistical analysis solely included recipients with ACR within the first three months after HTx compared to recipients without ACR in the first year. For biomarker evaluation a multicenter testing with a larger study cohort is necessary.

Previous studies characterized most of the used biomarkers as suitable markers for detection of ACR: percentage of CD25-positive T cells correlated with ACR after transplantation [15]. Intracellular IL-2 and IFN*γ* expression has been evaluated as surrogate marker to predict the risk of rejection in previous studies [16]. For the downstream chemokines of IFN*γ*-signalling, CXCL9 and CXCL10, it has been described that high pretransplant serum levels were predictive for ACR after organ transplantation [2, 3].

Our results revealed that percentage of pDCs changed according to anti-MICA-Ab positivity which in turn was associated with a lowered graft survival and higher acute rejection rates in previous studies [17]. Lower percentage of pDCs was detected in recipients with anti-MICA-Abs in our study. We conclude that in consequence of immunosuppressive therapy the number of pDCs was reduced after HTx but increased continuously during the first year after HTx.

A reduced number of pDCs in the early phase after HTx probably resulted in a reduced activation of regulatory T cells (T_regs_) through pDCs. One type of T_regs_, the IL-10-secreting type I T_regs_ (Tr1), has the ability to inhibit Ab production. Through this way, an association between the reduced percentage of pDCs and Ab positivity is conceivable. DCs seem to have a central role in monitoring patients with higher risk for rejection, especially in the early phase after HTx.

Furthermore, analysis of HLA and MICA sensitization showed that age and gender may influence results of biomarker analysis. Therefore, we recommend a stratified analysis for both parameters in the clinical validation process for biomarkers.

## 5. Conclusions

In summary, biological changes of biomarkers occur in the first year following HTx. The clinical validation of biomarkers is of great importance and should significantly influence data interpretation. A validation process should include (1) assessment of biological and statistical relevance to confirm a correlation between biomarker and clinical parameters like rejection and infection, (2) validation for different time points and different immunosuppressive drug therapies after transplantation, and (3) validation for cohorts of different compositions.

## Figures and Tables

**Table 1:** Overview about demographic data and transplantation history. Data are presented for the whole study population (row 2) and according to the appearance of rejection within the first year after HTx (rows 3 and 4).

	Whole population	Patients without rejection in the first year after HTx	Patients with rejection within the first 3 months after HTx
*n*	46	37	6
Age at HTx (±SD)	50.6 ± 12.5 yrs	50.5 ± 12.8 yrs	48.3 ± 11.3 yrs
Female gender (%)	*n* = 13 (28%)	*n* = 13 (35%)	*n* = 1 (17%)
Disease leading to HTx			
DCM	*n* = 23	*n* = 20	*n* = 3
ICM	*n* = 16	*n* = 12	*n* = 2
Other	*n* = 6	*n* = 5	*n* = 1
Assist device support before HTx	*n* = 5	*n* = 4	*n* = 1
Re-HTx	*n* = 1	*n* = 0	*n* = 1
Death within the first year after HTx	*n* = 2	*n* = 2	*n* = 0
Panel reactive antigen before HTx			
PRA = 0	*n* = 40	*n* = 33	*n* = 5
PRA > 0, <20	*n* = 3	*n* = 1	*n* = 1
PRA ≥ 20	*n* = 2	*n* = 2	*n* = 0
Transplant rejection (histopathological EMB grade ≤ grade IB or higher)			
1st quarter	*n* = 6	—	6
2nd quarter	*n* = 1	—	—
2nd half year	*n* = 2	—	—

DCM, dilated cardiomyopathy; EMB, endomyocardial biopsy; HTx, heart transplantation; ICM, ischemic cardiomyopathy; PRA, panel reactive antibody; Re-HTx, repeat heart transplantation; SD, standard deviation.

**Table 2:** Data sets with imputed values of the different immunological parameters CD25, CD95, IL2, IFN*γ*, plasmacytoid and myeloid dendritic cells, IP10, and MIG at different time points during the first year after heart transplantation. Data are represented as mean ± standard deviations.

	1st quarter	2nd quarter	2nd half year	Adjusted *p* value
CD25	33.6% ± 21.0%	34.7% ± 21.9%	33.4% ± 20.6%	0.78
CD95	39.2% ± 23.7%	46.3% ± 26.9%	45.6% ± 28.7%	0.22
IL-2	9.2% ± 11.6%	16.2% ± 13.8%	18.3% ± 15.5%	<0.01
IFN*γ*	10.7% ± 12.4%	17.9% ± 12.8%	24.9% ± 21.4%	<0.01
pDCs	18.8% ± 12.2%	26.4% ± 10.5%	31.0% ± 11.9%	<0.01
mDCs	41.2% ± 20.9%	49.7% ± 15.0%	47.7% ± 21.0%	0.06
pDC/mDC ratio	0.78 ± 1.17	0.71 ± 0.71	1.05 ± 1.22	0.24
IP10	42.1 pg/mL ± 42.0 pg/mL	35.1 pg/mL ± 25.5 pg/mL	45.5 pg/mL ± 43.9 pg/mL	0.25
MIG	29.6 pg/mL ± 38.3 pg/mL	23.7 pg/mL ± 16.6 pg/mL	25.4 pg/mL ± 13.6 pg/mL	0.40

CD25, alpha chain of the interleukin-2 receptor; CD95, Fas receptor; IFN*γ*, interferon g; IL2, interleukin-2; IP10, CXC chemokine ligand 10; mDCs, myeloid dendritic cells; MIG, CXC chemokine ligand 9; pDCs, plasmacytoid dendritic cells.

**Table 3:** Mean, standard deviations, and *p* values of the different immunological parameters CD25, CD95, IL2, IFN*γ*, plasmacytoid and myeloid dendritic cells, IP10, and MIG in recipients with and without acute cellular rejection at three different time points during the first year after heart transplantation. *p* values refer to the comparison of the two groups: recipients with acute cellular rejections and recipients without acute cellular rejections.

	1st quarter		2nd quarter		2nd half year	
	Mean ± SD	*p* value	Mean ± SD	*p* value	Mean ± SD	*p* value
CD25						
Without ACR	34.9% ± 20.7%	0.287	33.5% ± 21.0%	0.392	31.4% ± 20.1%	0.592
With ACR	22.7% ± 26.9%		44.1% ± 27.6%		37.2% ± 27.1%	
CD95						
Without ACR	42.2% ± 23.2%	0.033	43.6% ± 27.3%	0.288	42.8% ± 28.5%	0.510
With ACR	15.1% ± 19.2%		59.6% ± 23.8%		52.5% ± 29.4%	
IL-2						
Without ACR	9.9% ± 12.2%	0.537	15.2% ± 13.1%	0.150	18.0% ± 15.0%	0.130
With ACR	5.9% ± 9.1%		27.5% ± 16.6%		30.9% ± 16.9%	
IFN*γ*						
Without ACR	10.1% ± 11.1%	0.461	16.9% ± 12.2%	0.201	24.9% ± 21.5%	0.501
With ACR	19.3% ± 21.9%		27.2% ± 17.6%		33.2% ± 26.3%	
pDCs						
Without ACR	18.8% ± 12.4%	0.797	26.2% ± 11.4%	0.404	31.5% ± 12.3%	0.443
With ACR	17.1% ± 14.9%		32.0% ± 7.3%		36.6% ± 7.9%	
mDCs						
Without ACR	39.6% ± 20.2%	0.465	49.1% ± 15.9%	0.847	48.7% ± 21.8%	0.519
With ACR	48.2% ± 30.9%		51.0% ± 11.2%		40.5% ± 17.4%	
pDC/mDC ratio						
Without ACR	0.8 ± 1.3	0.702	0.7 ± 0.8	0.922	1.1 ± 1.3	0.915
With ACR	0.5 ± 0.4		0.7 ± 0.3		1.2 ± 0.8	
IP10						
Without ACR	36.5 pg/mL ± 33.4 pg/mL	0.289	29.9 pg/mL ± 21.6 pg/mL	0.017	39.8 pg/mL ± 38.7 pg/mL	0.511
With ACR	57.7 pg/mL ± 59.9 pg/mL		67.5 pg/mL ± 33.7 pg/mL		54.0 pg/mL ± 58.3 pg/mL	
MIG						
Without ACR	33.5 pg/mL ± 41.6 pg/mL	0.418	22.7 pg/mL ± 17.6 pg/mL	0.783	25.5 pg/mL ± 14.4 pg/mL	0.891
With ACR	16.1 pg/mL ± 7.8 pg/mL		26.3 pg/mL ± 11.8 pg/mL		24.5 pg/mL ± 12.0 pg/mL	

ACR, acute cellular rejection; CD25, alpha chain of the interleukin-2 receptor; CD95, Fas receptor; IFN*γ*, interferon g; IL-2, interleukin-2; IP10, CXC chemokine ligand 10; mDCs, myeloid dendritic cells; MIG, CXC chemokine ligand 9; pDCs, plasmacytoid dendritic cells; SD, standard deviation.
